# Minimally Invasive Sympathicotomy for Palmar Hyperhidrosis and Facial Blushing: Current Status and the Hyperhidrosis Expert Center Approach

**DOI:** 10.3390/jcm11030786

**Published:** 2022-01-31

**Authors:** Michiel Kuijpers, Judith E. van Zanden, Petra W. Harms, Hubert E. Mungroop, Massimo A. Mariani, Theo J. Klinkenberg, Wobbe Bouma

**Affiliations:** 1 Department of Cardiothoracic Surgery, University Medical Center Groningen, University of Groningen, 9700 RB Groningen, The Netherlands; j.e.van.zanden@umcg.nl (J.E.v.Z.); h.e.mungroop@umcg.nl (H.E.M.); m.mariani@umcg.nl (M.A.M.); t.j.klinkenberg@umcg.nl (T.J.K.); w.bouma@umcg.nl (W.B.); 2 Hyperhidrosis Expert Center, Dermatology, Martini Hospital, 9700 RM Groningen, The Netherlands; harmsp@mzh.nl

**Keywords:** facial blushing, palmar hyperhidrosis, sympathectomy, sympathicotomy, endoscopic thoracic sympathectomy (ETS), single port video-assisted thoracoscopic surgery (VATS), minimally invasive surgery

## Abstract

Hyperhidrosis, the medical term for excessive sweating beyond physiological need, is a condition with serious emotional and social consequences for affected patients. Symptoms usually appear in focal areas such as the feet, hands, axillae and face. Non-surgical treatment options such as topical antiperspirants or systemic medications are usually offered as a first step of treatment, although these therapies are often ineffective, especially in severe and intolerable cases of hyperhidrosis. In the treatment algorithm for patients suffering from hyperhidrosis, surgical thoracoscopic sympathicotomy offers a permanent solution, which is particularly effective in the treatment of palmar hyperhidrosis and facial blushing. In this review, we describe the current status of thoracoscopic sympathicotomy for palmar hyperhidrosis and facial blushing. In addition, we share the specific treatment approach, technique and results of our Hyperhidrosis Expert Center. Last, we share recommendations to ensure an effective, reproducible and safe application of single-port thoracoscopic sympathicotomy for palmar hyperhidrosis and facial blushing, based on our extensive experience.

## 1. Introduction

Hyperhidrosis or excessive sweating is a medical condition with disabling emotional and social consequences for affected patients [1]. The prevalence of primary hyperhidrosis has been estimated to be up to 5% in the population, and symptoms usually appear in focal areas such as the palms, axillae, feet or the face [2,3,4]. The pathophysiology of primary focal hyperhidrosis (PFH) has not yet been fully elucidated. A sympathetic hyperactivity of otherwise healthy eccrine sweat glands has been described, which may be triggered by stimuli such as emotions or heat. PFH should be distinguished from secondary hyperhidrosis, in which excessive sweating is usually more generalized and elicited by underlying systemic conditions or medication [5,6,7].

The field of treatment options for PFH is constantly evolving in an attempt to relieve patients of both mental and physical consequences of the disease. A step-wise approach with topical antiperspirants, botulinum toxin injections, iontophoresis and systemic medication is generally recommended [8,9,10]. Aluminum chloride has been reported as the most effective topical antiperspirant as a first-line treatment in axillary hyperhidrosis, and armpit dryness can be achieved within 48 h. However, an important disadvantage of this treatment is its transient nature of effect, with relapse of symptoms within 48 h after discontinuation of treatment [8]. Botulinum toxin injections are the most studied non-surgical treatment option for hyperhidrosis, and success rates of 76.5–81.4% have been described in a large multi-center trial on behalf of the hyperhidrosis study group. However, patients require repeated injections every 6–8 months to maintain effects [11]. Evidence for iontophoresis and systemic medication such as anticholinergics are limited and of low quality, although short-term benefits on hyperhidrosis symptoms were suggested in few studies [12]. The transient nature of these non-surgical treatment options may have non-satisfactory results due to the need for persistent adherence to therapy, especially in severe and intolerable cases of PFH [8,13,14]. Surgical treatment of PFH (sympathicotomy) offers a permanent solution and has proven to be particularly effective in palmar hyperhidrosis and facial blushing [14,15]. Therefore, in the treatment algorithm for patients suffering from severe or intolerable palmar hyperhidrosis or facial blushing, surgical treatment should be considered earlier [10,16,17]. The aim of this review is to share the current status of thoracoscopic sympathicotomy for palmar hyperhidrosis and facial blushing, and to share the specific treatment approach and results of the Hyperhidrosis Expert Center in Groningen, the Netherlands.

## 2. The Current Status of Minimally Invasive Sympathicotomy for Palmar Hyperhidrosis 

Sympathectomy was first described in 1889 by Alexander et al., who applied resection of the sympathetic chain and ablation of the ganglia as a treatment for epilepsy [18]. In 1919, Kotzareff et al. were the first to practice sympathectomy as a treatment for unilateral facial hyperhidrosis [19]. Adson et al. followed in 1935, by applying sympathectomy as a treatment for palmar hyperhidrosis [20]. Initially, sympathectomy was performed via open thoracotomy with relatively high morbidity rates [21]. The field of surgical treatment for PFH developed from an open approach to a much safer endoscopic approach (endoscopic thoracic sympathectomy, ETS), which was first described in 1975 [22]. Over the years, further refinements were made to optimize the endoscopic approach. The use of thoracoscopy equipment already enabled the use of small incisions, but the recent development of single-port thoracoscopy over more conventional bi- or tri-portal approaches decreased the degree of invasiveness even further [23].

Currently, endoscopic surgery is considered an effective treatment for moderate to severe palmar hyperhidrosis [23]. Success rates of over 90% for curing or improving symptoms of severe palmar hyperhidrosis have been described [24,25,26,27,28]. More recent studies by Chen et al. and Feng et al., in which respectively 10.275 and 200 patients with palmar hyperhidrosis were treated with thoracoscopic sympathicotomy, even reported a success rate of 100% [29,30]. Nevertheless, literature comparisons should be interpreted with caution, since in most studies different nomenclature and definitions are used. 

First, different techniques for targeting the sympathetic chain are practiced, such as sympathectomy versus sympathicotomy. In the case of sympathectomy, both the sympathetic ganglia and chain are destroyed and partially removed. However, in the case of sympathicotomy, the sympathetic chain is only transected [31]. A comparative meta-analysis by Du et al. showed that the satisfaction of patients that underwent sympathectomy versus sympathicotomy were comparable, which is in line with earlier studies [32,33,34]. It is hypothesized that severing of the sympathetic reflex arcs, which run through the ganglia to the hypothalamus, leads to a higher incidence of compensatory hyperhidrosis (CH) through dysfunctional sweat regulation in the affected body parts [33,35,36]. These findings strengthen the belief that the ganglia should be left untouched and that sympathicotomy should therefore be preferred over sympathectomy. In addition, sympathicotomy is associated with shorter operating times and is considered a more feasible technique [37].

Second, besides differences in the nomenclature for techniques of targeting the sympathetic chain, the level of interruption is described with various definitions. Some studies describe the vertebral level of nerve interruption, while others refer to rib levels. As a consequence, literature comparisons of postoperative treatment success rates and complication rates are difficult to make. For that reason, the Society of Thoracic Surgeons suggested in their expert consensus to adopt an international nomenclature that refers to rib levels (R) as anatomic landmarks, instead of the vertebral level at which the nerve is interrupted [38]. As for patients with isolated palmar hyperhidrosis, interruption of the sympathetic nerve at the level of R3 is recommended [38]. Other studies suggest interruptions at both levels R3 and R4 to achieve completely dry hands. Nevertheless, the risk of developing CH, a common side effect associated with ETS, is higher in these patients. Besides these side effects, major complications may occur, such as intraoperative bleeding, pneumothorax and infections. To minimize perioperative risks, adequate screening and selection of patients is essential. When complications occur, they should be adequately managed by experienced teams, and surgery should be carried out by surgeons trained in minimally invasive techniques [39]. 

## 3. The Hyperhidrosis Expert Center Approach of Minimally Invasive Sympathicotomy for Palmar Hyperhidrosis: Selection, Technique and Results

### 3.1. Selection of Patients for Minimally Invasive Sympathicotomy for Palmar Hyperhidrosis

ETS for palmar hyperhidrosis patients offers a high percentage of success and satisfaction; however as stated earlier, the importance of thorough pre-operative counseling cannot be stressed enough [16]. This counseling should be aimed at selecting the right patients for the procedure and for managing expectations, which includes (but is not limited to) providing information on the possibility of attaining a quite variable degree of CH postoperatively. CH is often mistakenly seen as a complication instead of a side effect of the procedure. In the Hyperhidrosis Expert Center, only hyperhidrosis patients suffering from PFH, as defined by Hornberger et al. (Table 1), are considered for surgery [40]. Various types of secondary hyperhidrosis warrant further medical examination [40]. To quantify the impact of PFH on the daily activities of a potential surgical candidate, the Hyperhidrosis Disease Severity Scale (HDSS) is used (Table 2). Only patients rating the severity of their PFH as severe (HDSS 3) or intolerable (HDSS 4) qualify for surgery. Due to a higher recurrence rate of PFH in children younger than 17 years of age, an HDSS score of 4 is mandatory before surgery is even considered in children [41].

### 3.2. Surgical Technique of Minimally Invasive Sympathicotomy for Palmar Hyperhidrosis

In 2010 a ‘new idea’ was born to use the VasoView^®^ device (Maquet Inc, Rastatt, Germany) to further minimize the invasiveness of thoracoscopic sympathicotomy in the treatment of palmar hyperhidrosis [42]. This novel technique initiated a state-of-the-art, reproducible, and safe single-port procedure with excellent short- and long-term results [16,23].

**Figure 1:**
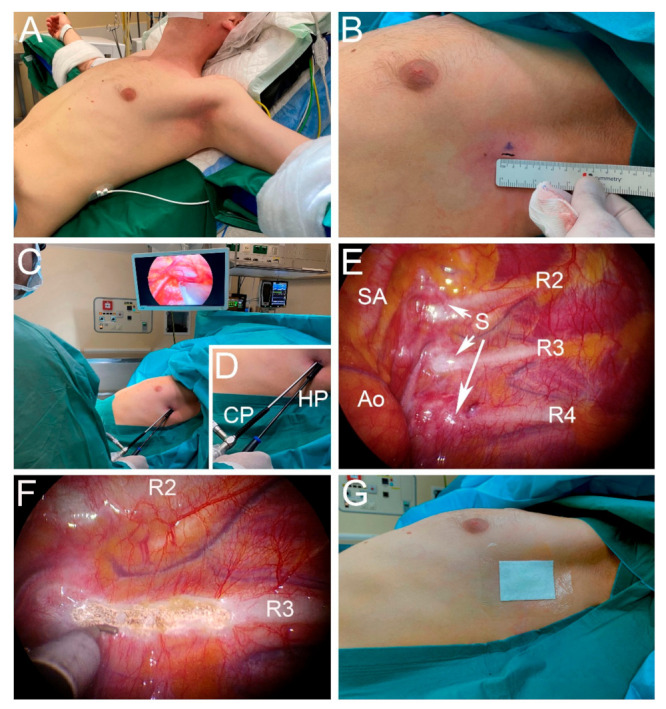
Surgical technique of single-port R3 sympathicotomy for palmar hyperhidrosis. (**A**) Patient positioned in the ‘beach chair’ or semi-Fowler position, seated at a 45^o^ angle, with both arms spread out. (**B**) Marked incision directly posterior to the axillary/mammary fold. (**C**) The camera and cautery hook are inserted through the same incision, over a camera port and hook port. (**D**) Close-up of the camera port (CP) and hook port (HP). (**E**) Thoracoscopic view of the left superior mediastinum. Identification of the subclavian artery (SA), aorta (Ao), sympathetic nerve (S) and the second (R2), third (R3) and fourth ribs (R4). (**F**) Thoracoscopic view after R3 sympathicotomy. The sympathetic chain is transected on the mid-rib level in an attempt to spare the ganglia. (**G**) Double plastic-coated sticker placed over the wound, preventing air re-entry.

The operation is performed under general anesthesia. Patients are intubated using a standard single lumen tube. No double lumen tube is required since the operation itself is performed under apnea. The patient is ventilated with 100% FiO_2_, which allows for a quicker collapse of the lung and prolongs the time that the patient can safely be held in apnea. Patients are positioned in the ‘beach-chair’ or semi-Fowler position, seated at a 45° angle, with both arms spread out (Figure 1A). This position does not only allow for comfortable bilateral access during the surgical procedure without the need to move an anesthetized patient, but it facilitates full view of the apical intrathoracic operative field due to the fact that gravity forces the collapsed lung dorso-caudally. 

After the patient is prepped and draped, the procedure is first conducted on the right side. The incision area is infiltrated with 5 mL of bupivacaine 0.25% (+ adrenalin, which reduces the chance of bleeding and subcutaneous hematoma formation) from skin to costal periosteum. A 7 mm wide incision in the skin lines is made, directly posterior to the axillary/mammary fold (Figure 1B). Next, blunt flush supracostal preparation is performed with a small clamp to and through the intercostal muscles, taking care not to perforate the parietal pleura at this stage. Apnea is requested with a fully disconnected tube, which enables efflux of air from the lungs, favoring lung collapse. A blunt 7 mm trocar is used for entry through the parietal pleura into the thoracic cavity, while making sure that the entry is directly over the rib. For this procedure, we use a slender 7 mm ribbed hard-plastic work-port. More advanced ports that include a CO_2_ port with a one-way valve have the following three disadvantages when using single-port access: First, these trocars are designed to be as occlusive as possible, not allowing for free flow of air in both directions. The blockage of free flow prevents a quick influx of air and thus hinders lung collapse. Second, the thick ‘head’ of the trocar aggravates the competition of surgical instruments that one is trying to align through the same incision. Third, a cautery hook with CO_2_ insufflation at the tip not only solves the non-CO_2_-trocar problem, but also enables the surgeon to use the stream of CO_2_ to clear the operative field when minor bleeding needs to be controlled. The 5 mm camera is inserted through the work-port. This work-port is then pulled back over the camera shaft, and a new work-port is inserted directly adjacent to the bare camera shaft (Figure 1C,D). In this phase, we prevent damaging intrathoracic structures by actively freezing the camera position, taking care to not inadvertently advance the camera while inserting the second port. After removal of the trocar, the unobstructed inflow of air through the open work-port will quickly facilitate enough collapse of the lung for full view of the intrathoracic apical operative field and correct for identification of the rib levels and sympathetic nerve (Figure 1E). In our experience, CO_2_ insufflation is only needed when pleural adhesions are prominent, or in some patients requiring R3-R5 sympathicotomy for axillary or combined palmar/axillary hyperhidrosis in which collapse of the lung alone impedes proper access to the fifth rib. Subsequently, a small caliber cautery hook is inserted through the work-port (Figure 1C,D). Sympathicotomy is performed on the mid-rib level, providing a firm background for thorough cautery, by using low-energy settings to avoid thermal spread that may damage neighboring ganglia. This transection is extended by 2 cm laterally over the rib to capture more spread-out variations in anatomy. An active search for nerve of Kuntz is undertaken, and such nerves are divided when present (Figure 1F). After removal of the cautery hook, an 8 Fr. silicone drain is inserted through the work-port and advanced to the apex of the thoracic cavity under direct vision. The camera is removed, and the drain ensures adequate drainage of residual air while the lung is recruited and re-insufflated by manual ventilation for 2 min. In the meantime, a subcutaneous purse-string suture is placed around the drain. After 2 min of recruiting, the drain is removed under inspiratory hold of 30 cm H_2_O. The subcutaneous purse string is knotted, sealing the intra-thoracic vacuum, and normal ventilation is started. The skin is closed intracutaneously. For redundancy, a double plastic-coated sticker is placed over the wound, again preventing air re-entry (Figure 1G). After skin-closure on the right side, a left-sided procedure is performed in an identical fashion.

Postoperatively, a chest X-ray is taken to exclude a significant residual pneumothorax and other possible complications. As the Hyperhidrosis Expert Center takes many referrals from outside the local region, patients are kept in the hospital overnight and are discharged the next morning. As for the pain regimen, patients are treated with paracetamol 1000 mg four times per day, and diclofenac 50 mg three times per day for the first week. Nevertheless, most patients do not require analgesics for longer than 3 days. Follow-up consists of a telephone interview 2 weeks after surgery. Inquiries are made regarding obtained effect, current HDSS score, postoperative pain, and presence and severity of CH. 

### 3.3. Results of the Hyperhidrosis Expert Center Approach of Minimally Invasive Sympathicotomy for Palmar Hyperhidrosis

As of 2021, over 1500 procedures with the technique we previously described have been performed in the Hyperhidrosis Expert Center. Recently, we published the results of 326 prospectively analyzed procedures [16]. Demographics of the operated patient population were as follows: 52.8% of patients were female, mean age was 30.9 ± 9.8 years and mean Body Mass Index was 24.0 ± 3.6 kg/m^2^. Patients had no relevant medical history. Improvement scores for hyperhidrosis two weeks after the procedure were excellent, since almost all patients experienced a ≥2 point reduction in HDSS score. After 18 months of follow-up 68.7% of patients still experienced a ≥2 point reduction in HDSS score, and 22.7% of the patients still had a reduction of 1 point in HDSS score. Quality of life significantly improved after 18 months of follow-up. Development of CH was absent or moderate in 70.6% of patients, and severe in 29.4%. No major complications, such as bleeding, conversion to thoracotomy or death were observed. Residual pneumothorax occurred in 1.8%. Overall recommendation rate of the procedure, queried as ‘would you recommend the procedure, considering the obtained effect’ was 87.7% [16].

## 4. The Current Status of Minimally Invasive Sympathicotomy for Facial Blushing

The success of ETS as a treatment for patients suffering from palmar hyperhidrosis led to the idea to extend the application of this technique to patients suffering from facial blushing. In these patients, excessive facial redness occurs in response to emotional stimuli. As a consequence, patients may experience feelings of embarrassment, depression and social anxiety [43]. The exact prevalence of facial blushing is unknown, but it is reported as a prominent symptom in 50% of patients with social anxiety disorder, with a prevalence of around 10% in the general population [44,45].

Treatment options for facial blushing are limited. Selective serotonin reuptake inhibitors (SSRI’s) are suggested to reduce facial blushing and social phobia; however, only a few studies specifically address the effect on symptoms of facial blushing itself [46,47]. Beta-receptor blockers are prescribed as well, although their effectiveness is mostly anecdotal, and evidence for the application in facial blushing is lacking [48]. In addition to medical treatment, small-scale studies suggest successful application of psychological therapy such as cognitive–behavioral therapy to improve coping mechanisms for fear of blushing [49,50]. Nevertheless, psychological treatment is often time-consuming and transient of nature [46]. Therefore, ETS for facial blushing is suggested as an attractive, permanent solution. In an early retrospective study of Drott et al. in which 831 patients were included, relief of blushing was described in 94% of patients [48]. A later retrospective study by Licht et al. reported relief of blushing in 74% of patients, and an additional 16% of patients experienced partial relief of blushing [51]. In 2017, Girish et al. included the aforementioned studies in a systematic review of nine studies and analyzed a total of 1369 patients [52]. Overall relief of blushing was reported in 78,30% of patients, and complete satisfaction was obtained in 84,02% of patients treated with ETS [52]. These studies show that thoracoscopic sympathicotomy may offer a high percentage of success and satisfaction, although percentages of success may vary between centers [45,52,53,54].

As described earlier for palmar hyperhidrosis, the level of nerve interruption for facial blushing differs between studies as well. For facial blushing, nerve interruption is currently recommended at the level of R2 [38,55]. Earlier studies recommended nerve interruption at a R2-R3 level, but this comes with more side effects and higher CH rates [51]. Possible complications in patients treated with ETS for facial blushing are the same as in palmar hyperhidrosis, such as intraoperative bleeding, pneumothorax and infections. In addition, the possibility of developing Horner’s syndrome and gustatory sweating should be closely observed in patients treated for facial blushing, since nerve interruption at level R2 may lead to collateral damage to the stellate ganglion [8,45,52,54]. The incidence of Horner’s syndrome after ETS for facial blushing varies between 0% and 4% [8,45,51,52], although this complication is probably often related to the used operative technique and experience of the surgeon. The reproducible and safe single-port sympathicotomy procedure of the Hyperhidrosis Expert Center was considered as a perfect approach for the treatment of these patients. Our short- and long-term success rates and expertise in the treatment of palmar hyperhidrosis with over 1500 thoracoscopic procedures in the last decade prompted us to broaden the scope to treatment of patients with severe facial blushing [16,23].

## 5. The Hyperhidrosis Expert Center Approach of Minimally Invasive Sympathicotomy for Facial Blushing: Selection, Technique and Results 

### 5.1. Selection of Patients for Minimally Invasive Sympathicotomy for Facial Blushing

Patient selection and thorough pre-operative counseling are of paramount importance, especially in patients with severe facial blushing. The type of blushing should be carefully questioned. The only kind of blushing that responds well to sympathicotomy is the facial rapid onset type that appears in seconds, and it is associated with embarrassment and the urge to flee from a situation [45,54]. The facial blushing must have a severe impact on the daily quality of life, which is a crucial fact in accepting a patient for sympathicotomy. In addition, pharmacological and physiological regimens must have proven ineffective. Preoperative counseling is of paramount importance, because of a higher risk of side effects. The surgeon should guide the patient through an informed and wise selection of treatment options [45,56,57,58,59,60]. In addition, the patient should receive a thorough disclosure of all advantages and disadvantages of sympathicotomy to ensure a realistic expectation of success, side effects and complications. Patients should be made aware that sympathicotomy is essentially irreversible [61]. The complication rate can be considered marginal and as low as in the thoracoscopic treatment for palmar hyperhidrosis, since the same technique is used with the exception of the level of nerve interruption [16,23,45]. 

### 5.2. Surgical Technique for Minimally Invasive Sympathicotomy for Facial Blushing

In addition to patient selection, surgeon selection is also important [54]. The surgeon should have adequate experience in video-assisted thoracoscopy and the capacity to deal with potential intraoperative complications. The surgical technique for severe facial blushing is mainly identical to the surgical technique for palmar hyperhidrosis, as described above. The only two differences are *the level* of sympathicotomy, which is performed at the R2 level instead of R3 level, and a *staged* single-port thoracoscopic procedure. This staged approach means that thoracoscopic surgery is first performed unilaterally, possibly followed by a contralateral procedure after 4–6 weeks. The design of staged surgery is deliberately chosen for several reasons. First, there is a higher risk of Horner’s syndrome because we operate at level R2. In case of unilateral Horner’s syndrome, a staged procedure allows for cancellation of the contralateral procedure to avoid possible bilateral Horner’s syndrome. Second, if the procedure is not successful on one side, contralateral sympathicotomy is not considered useful. Third, staged surgery may reduce the risk of CH [62,63]. Bilateral sympathicotomy at level R2 increases the risk of CH, as shown in the literature [53,54,64].

The same as for patients operated for palmar hyperhidrosis, patients are kept in the hospital overnight and are discharged the next morning, since the Hyperhidrosis Expert Center takes many referrals from outside the local region. Likewise, the pain regimen is the same as for patients treated for palmar hyperhidrosis, as described above. Follow-up of patients consists of a telephone interview 2 weeks after surgery. Inquiries are made regarding obtained effect, postoperative pain, and presence and severity of CH and gustatory sweating. When the patient is satisfied with the obtained effect, a contralateral sympathicotomy is scheduled.

### 5.3. Results of the Hyperhidrosis Expert Center Approach for Minimally Invasive Sympathicotomy for Facial Blushing

As of 2021, we have performed more than 40 single-stage single-port thoracoscopic R2 sympathicotomy procedures for severe facial blushing in the Hyperhidrosis Expert Center. Demographics of the operated patient population were as follows: 36% of patients were female, mean age was 33 ± 9.2 years and mean Body Mass Index was 24 ± 4.6 kg/m^2^. Patients had no relevant medical history. A preliminary analysis after a mean follow-up time of 25 months suggests excellent results, with patient satisfaction scores of 100%. One patient experienced transient Horner’s syndrome, which resolved within one week after surgery (unpublished data). 

## 6. Recommendations and Conclusions

To ensure an effective, reproducible and safe application of single-port thoracoscopic sympathicotomy for palmar hyperhidrosis or facial blushing, we would like to make the following recommendations: First, adequate and thorough patient selection is crucial for success. Second, a truly experienced surgeon performing high volumes of thoracoscopic surgery in a team that is skilled in treating possible complications is key to success; quality comes with quantity. Last, we firmly believe that single-port thoracoscopic sympathicotomy should be offered to patients suffering from severe palmar hyperhidrosis or severe facial blushing at an earlier stage of the treatment algorithm [16]. With these recommendations in mind, we would like to conclude that single-port thoracoscopic sympathicotomy is an effective and safe treatment option in carefully selected patients suffering from severe palmar hyperhidrosis and severe facial blushing.

## Figures and Tables

**Table 1:** Criteria for establishing PFH as recommended by Hornberger et al. [40].

Focal, Visible, Excessive Sweating of at Least 6 Months Duration Without Cause with at Least Two of the Following Characteristics:	
1	Bilateral and relatively symmetric
2	Impairs daily activities
3	Frequency of at least one episode per week
4	Age of onset less than 25 years
5	Positive family history
6	Cessation of focal sweating during sleep

**Table 2:** Hyperhidrosis Disease Severity Scale (HDSS). Only patients rating the severity of their PFH as severe (HDSS 3) or intolerable (HDSS 4) qualify for surgery.

HDSS Score	‘How Would You Rate the Severity of Your Sweating’	PFH Severity
1	‘My sweating is never noticeable and never interferes with my daily activities’	Mild
2	‘My sweating is tolerable but sometimes interferes with my daily activities’	Moderate
3	‘My sweating is barely tolerable and frequently interferes with my daily activities’	Severe
4	‘My sweating is intolerable and always interferes with my daily activities’	Intolerable

## Data Availability

Not applicable.
